# Design, Synthesis, and Biological Evaluation of Novel Biotinylated Podophyllotoxin Derivatives as Potential Antitumor Agents

**DOI:** 10.3389/fchem.2019.00434

**Published:** 2019-06-18

**Authors:** Cheng-Ting Zi, Ying-Sheng Gao, Liu Yang, Shu-Yun Feng, Yue Huang, Li Sun, Yi Jin, Feng-Qing Xu, Fa-Wu Dong, Yan Li, Zhong-Tao Ding, Jun Zhou, Zi-Hua Jiang, Sheng-Tao Yuan, Jiang-Miao Hu

**Affiliations:** ^1^State Key Laboratory of Phytochemistry and Plant Resources in West China, and Yunnan Key Laboratory of Natural Medicinal Chemistry, Kunming Institute of Botany, Chinese Academy of Sciences, Kunming, China; ^2^Jiangsu Key Laboratory of Drug Screening and Jiangsu Center for Pharmacodynamics Research and Evaluation, China Pharmaceutical University, Nanjing, China; ^3^Key Laboratory of Pu-er Tea Science, College of Science, Ministry of Education, Yunnan Agricultural University, Kunming, China; ^4^Key Laboratory of Medicinal Chemistry for Nature Resource, School of Chemical Science and Technology, Ministry of Education, Yunnan University, Kunming, China; ^5^Department of Chemistry, Lakehead University, Thunder Bay, ON, Canada

**Keywords:** podophyllotoxin derivatives, biotin, anticancer activity, synthesis, apoptosis

## Abstract

Podophyllotoxin has long been used as an active substance for cytotoxic activity. Fourteen novel biotinylated podophyllotoxin derivatives were designed, synthesized, and evaluated for cytotoxic activity for this study. The synthesized compounds were evaluated for cytotoxic activity in the following human cancer cell lines, SW480, MCF-7, A-549, SMMC-7721, and HL-60 by MTT assay. Most of them exhibited potent cytotoxic effects and compound **15** showed the highest cytotoxic activity among the five cancer cell lines tested, having its IC_50_ values in the range of 0.13 to 0.84 μM. Apoptosis analysis revealed that compound **15** caused obvious induction of cell apoptosis. Compound **15** significantly down-regulated the expression level of the marker proteins (caspase-3 and PARP) in H1299 and H1975 cells, activated the transcription of IRE1α, increased the expression of GRP78 and XBP-1s, and finally induced apoptosis of H1299 cells. *In vivo* studies showed that **15** at a dose of 20 mg/kg suppressed tumor growth of S180 cell xenografts in icr mice significantly. Further molecular docking studies suggested that compound **15** could bind well with the ATPase domain of Topoisomerase-II. These data suggest that compound **15** is a promising agent for cancer therapy deserving further research.

## Introduction

Cancer is a kind of frequently-occurring disease that seriously threatens human health. In recent years, more attention has been focused on targeting anti-cancer drugs. Development of targeted antitumor drugs, increase of bioavailability and decrease of toxicity are the key topics which are currently being studied. Research efforts in these topics have already led to the discovery of new drug leads and molecular scaffolds important for the development of novel antitumor agents (Fulda, 2010; Qiao et al., 2011; Wen et al., 2012). Currently, targeted cancer therapy has attracted a lot of interests in cancer research and has emerged as a new treatment option for various types of cancers.

Natural compounds are valuable sources with various structures, unique biological activities, and specific selectivity. Natural products have served as important sources of lead compounds for antitumor agents which have been developed for clinical use. However, many potential drugs lack tumor selectivity and often display significant toxic side effects, which hampers their development for clinical use (Holschneider et al., 1994; Bermejo et al., 2005). In order to enhance the therapeutic specificity of anticancer drugs, various targeting strategies have been explored, including antibodies (Wu and Ojima, 2004; Schrama et al., 2006; Lambert and Berkenblit, 2018), nanocarriers (Peer et al., 2007; Bonifácio et al., 2014; Hojjat-Farsangia et al., 2014; Senthilkumar et al., 2015), peptides (Mastrobattista et al., 1999; Dharap et al., 2005), and vitamins (Sawant et al., 2008; Chen et al., 2010; Guaragna et al., 2012). In each case, molecular features overexpressed on cancer cells are being targeted.

It has been widely recognized that all living cells depend on vitamins for survival and growth and obviously cancer cells must require higher amount of vitamins to meet the need of their rapid growth. Consequently, in order to sustain their rapid cell growth and enhanced proliferation, many cancer cells over-express receptors for certain vitamins. Therefore, vitamin receptors on the surface of these cells are important biomarkers for the delivery of tumor-targeted drugs (Russell-Jonesa et al., 2004; Leamon, 2008; Lu and Low, 2012). Biotin (vitamin H) is a nutrient required for cell growth, and tumor cells need substantially higher amounts of biotin than normal cells due to their rapid growth (Russell-Jonesa et al., 2004). Recent studies have shown that many cancer cell lines express higher levels of biotin receptors (BRs) than normal cells, e.g., L1210FR (leukemia), Ov2008 (ovarian), Colo-26 (colon), P815 (mastocytoma), M109 (lung), RENCA (renal), and 4T1 (breast) cancer cell lines (Russell-Jonesa et al., 2004; Chen et al., 2010). Thus, BR has emerged as a useful biomarker for targeted delivery of anti-tumor agent, and biotin as a tumor-targeting module has been successfully employed for the construction of small molecule antitumor drug conjugates (Chen et al., 2008; Ojima, 2008; Ojima et al., 2012).

**Figure 1:**
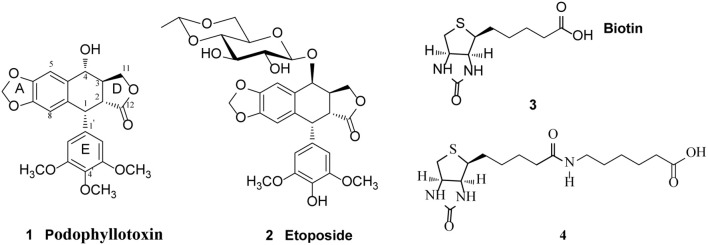
Structure of Podophyllotoxin **(1)**, Etoposide **(2)**, Biotin **(3)**, and 6-biotinylaminocaproic acid **(4)**.

The natural lignan podophyllotoxin (**PPT**, **1**, Figure 1) is isolated from *Dysosma versipellis* and shows cytotoxic activity against a variety of cancer cell lines by inhibiting microtubule assembly. However, **PPT** lacks tumor specificity and its high toxicity toward normal cells prevents its use in clinic for cancer treatment (Jardine, 1980; Desbene and Giorgi-Renault, 2002; Liu et al., 2007). The biological activity of **PPT** has led to extensive structural modification, resulting in several clinically useful compounds including etoposide (**2**, Figure 1), a semisynthetic glucosidic cyclic acetal of **PPT**. Etoposide exerts cytotoxic activity by inhibiting DNA topoisomerase II and the discovery of its novel mechanism of action led to further studies on the structure-activity relationship of **PPT** derivatives resulted from the structural modification at the C-4-position (Reddy et al., 2008; Zhang et al., 2014). Studies have shown that improvement in topoisomerase II inhibitory activity, water solubility, cytotoxic activity, drug resistance profile, and antitumor spectra of this class of compounds might be achieved through rational modification at C-4 position (Bromberg et al., 2003).

With the aim to improve the therapeutic efficacy and reduce the toxic side effects of podophyllotoxin in the treatment of cancer, we have designed a group of biotin-podophyllotoxin (Bio-PT) conjugates by covalently linking a biotin residue to podophyllotoxin. Such Bio-PT conjugates are anticipated to be taken up by cells through receptor-mediated endocytosis and selective delivery of these conjugates to cancer cells may be achieved due to a higher level of biotion receptors expressed on cancer cells. Here we report the synthesis of 14 biotinylated podophyllotoxin derivatives and their anticancer activity against various cancer cell lines. The compound with the highest anticancer activity was further studied to reveal the anticancer mechanisms and its antitumor effect was evaluated through *in vivo* studies as well.

## Materials and Methods

### General Information

All cancer cells were obtained from a Shanghai cell bank in China. All reagents were commercially available and were used without further purification unless otherwise indicated. Podophyllotoxin was purchased from Shanghai Yuanye Bio-Technology Co., Ltd (Shanghai, China). Anhydrous solvents were obtained by distillation from the indicated systems immediately prior to use: dichloromethane from calcium hydride and tetrahydrofuran from sodium. Uncorrected melting points were measured on a Beijing Taike XT-4. Electrospray ionization mass spectrometry (ESI-MS) data were acquired on API Qstar Pulsar instrument; High resolution electrospray ionization mass spectrometry (HRESI-MS) data were obtained on LCMS-IT-TOF (Shimadzu, Kyoto, Japan); All NMR spectra were recorded with Bruker AV-400 or DRX-500 or Bruker AVANCE III-600 (Bruker BioSpin GmbH, Rheinstetten, Germany) instruments, with tetramethylsilane (TMS) as an internal standard: chemical shifts (δ) are given in ppm and coupling constants (*J*) in Hz. Column chromatography (CC) are carried out using silica gel (200–300 mesh; Qingdao Makall Group CO., LTD; Qingdao; China). All reactions were monitored by analytical thin-layer chromatography (TLC), which was visualized by ultraviolet light (254 nm) and/or 10% phosphomolybdic acid/EtOH.

### Synthesis

#### General Procedure for the Preparation of Biotinylated Podophyllotoxin Derivatives 13–26

N,N′-diisopropylcarbodiimide (DIC, 0.6 mmol) and 4-dimethylaminopyridine (DMAP, 0.2 mmol) were added to a solution of biotin or 6-biotinylaminocaproic acid (0.2 mmol), podophyllotoxin or its derivative (0.2 mmol) in N, N-dimethylformamide (DMF, 2.5 mL). The reaction mixture was stirred at room temperature for 24 h under N_2_. Solvents were removed under reduced pressure. The residue was purified by chromatography over silica gel (CHCl_3_/CH_3_OH = 9:1) to afford the desired product.

### 4α-(biotin)-4-deoxypodophyllotoxin 13

White amorphous powder, yield 64%; m.p. 210–215°C; ${\left[\alpha \right]}_{\text{D}}^{26.2}$: +34.6 (*c* 0.13, DMSO); ^1^H-NMR (C_2_D_6_SO, 500 MHz) δ 6.83 (s, 1H, C^5^-H), 6.60 (s, 1H, C^8^-H), 6.52 (s, 2H, C^2^′, C^6^′-H), 6.01–5.98 (m, 2H, OCH_2_O), 5.67 (d, 1H, *J* = 6.0 Hz, C^4^-H), 4.46–4.42 (m, 1H), 4.29 (d, 1H, *J* = 4.5 Hz, C^1^-H), 4.27–4.23 (m, 2H), 4.10–4.04 (m, 1H), 3.70 (s, 6H, C^3^′, C^5^′-OCH_3_), 3.62 (s, 3H, C^4^′-OCH_3_), 3.05–3.01 (m, 1H), 2.79 (dd, 1H, *J* = 4.5Hz, 11.5 Hz, C^2^-H), 2.57–2.54 (m, 2H), 2.43–2.40 (m, 1H, C^3^-H), 2.16 (t, 2H, *J* = 9.0 Hz, C^8^^‴^-CH_2_), 1.58–1.52 (m, 2H), 1.42–4.40 (m, 2H), 1.26–1.21 (m, 2H); ^13^C-NMR (C_2_D_6_SO, 100 MHz) δ 177.5 (C-12), 172.6 (C-7^‴^), 162.8 (C-16^‴^), 152.7 (C-3′, C-5′), 147.5 (C-7), 146.3 (C-6), 138.4 (C-1′), 136.0 (C-4′), 132.4 (C-9), 126.8 (C-10), 109.2 (C-5), 108.2 (C-8), 105.6 (C-2′, C-6′), 101.3 (OCH_2_O), 72.1 (C-11), 70.5 (C-4), 61.0 (C-13^‴^), 60.0 (C-14^‴^), 59.2 (4′-OCH_3_), 55.9 (3′, 5′-OCH_3_), 55.3 (C-12^‴^), 43.6 (C-2), 43.5 (C-1), 40.1 (C-15^‴^), 38.9 (C-3), 33.4 (C-8^‴^), 28.0, 27.9, 24.4; ESIMS: m/z 641 [M + H] ^+^, HRESIMS: calcd for C_32_H_36_N_2_O_10_SH [M + H]^+^ 641.2138, found 641.2163.

### 4α-(6-biotinylaminocaproic acid)-4-deoxypodophyllotoxin 14

White amorphous powder, yield 54%; m.p. 147–149°C; ${\left[\alpha \right]}_{\text{D}}^{26.0}$: −14.6 (*c* 0.10, Pyridine); ^1^H-NMR (CDCl_3_, 400 MHz) δ 6.72 (s, 1H, C^5^-H), 6.50 (s, 1H, C^8^-H), 6.35 (s, 2H, C^2^′, C^6^′-H), 5.96 (d, 2H, *J* = 4.0 Hz, OCH_2_O), 5.85 (d, 1H, *J* = 9.2 Hz, C^4^-H), 4.57 (d, 1H, *J* = 4.0 Hz, C^1^-H), 4.50–4.48 (m, 1H), 4.34–4.27 (m, 2H), 4.17 (t, 1H, *J* = 9.6 Hz), 3.77 (s, 3H, C^4^′-OCH_3_), 3.73 (s, 6H, C^3^′, C^5^′-OCH_3_), 3.19–3.12 (m, 2H, C^12^^‴^-H, C^3^-H), 2.94–2.89 (m, 1H, C^2^-H), 2.88–2.84 (m, 2H), 2.79–2.69 (m, 2H), 2.40 (t, 2H, *J* = 7.2 Hz, C^2^^‴^-H), 2.18 (t, 2H, *J* = 6.8 Hz, C^8^^‴^-CH_2_), 1.68–1.64 (m, 4H), 1.51–1.49 (m, 2H), 1.40–1.35 (m, 2H), 1.31–1.29 (m, 2H), 1.18–1.17 (m, 2H); ^13^C-NMR (CDCl_3_, 100 MHz) δ 174.1 (C-12), 173.8 (C-1^‴^), 173.5 (C-7^‴^), 164.1 (C-16^‴^), 152.5 (C-3′, C-5′), 148.1 (C-7), 147.5 (C-6), 136.9 (C-1′), 134.9 (C-4′), 132.2 (C-9), 128.3 (C-10), 109.7 (C-5), 108.0 (C-2', C-6'), 106.9 (C-8), 101.6 (OCH_2_O), 73.4 (C-11), 71.3 (C-4), 61.8 (C-13^‴^), 60.7 (4′-OCH_3_), 60.3 (C-14^‴^), 56.1 (3′, 5′-OCH_3_), 55.7 (C-12^‴^), 45.4 (C-2), 43.6 (C-1), 40.5, 39.2, 38.7, 35.8, 34.1, 29.2, 28.2, 28.0, 26.4, 25.3, 24.5; ESIMS: *m/z* 789 [M + Cl]^−^, HRESIMS: calcd for C_38_H_47_N_3_O_11_SCl [M + Cl]^−^ 789.2625, found 789.2625.

### 4β-(biotin)-4-deoxypodophyllotoxin 15

White amorphous powder, yield 65%; m.p. 110–112°C; ${\left[\text{α}\right]}_{\text{D}}^{26.9}$: −51.6 (*c* 0.21, CHCl_3_); ^1^H-NMR (CDCl_3_, 400 MHz) δ 6.86 (s, 1H, C^5^-H), 6.54 (s, 1H, C^8^-H), 6.26 (s, 2H, C^2^′, C^6^′-H), 6.14 (d, 1H, *J* = 3.2 Hz, C^4^-H), 5.98–5.96 (m, 2H, OCH_2_O), 4.66 (d, 1H, *J* = 4.8 Hz, C^1^-H), 4.52–4.49 (m, 1H), 4.36–4.28 (m, 2H), 3.88 (t, 1H, *J* = 8.8 Hz), 3.79 (s, 6H, C^3^′, C^5^′-OCH_3_), 3.73 (s, 3H, C^4^′-OCH_3_), 3.24 (dd, 1H, *J* = 5.2 Hz, 14.4 Hz), 3.15–3.10 (m, 1H), 2.99–2.96 (m, 1H), 2.89 (dd, 1H, *J* = 4.8 Hz, 12.8 Hz, C^2^-H), 2.75–2.71 (m, 2H), 2.38 (t, 2H, *J* = 7.2 Hz, C^8^^‴^-CH_2_), 1.71–1.66 (m, 4H), 1.45–1.43 (m, 2H); ^13^C-NMR (CDCl_3_, 100 MHz) δ 174.2 (C-12), 173.3 (C-7^‴^), 163.7 (C-16^‴^), 152.6 (C-3′, C-5′), 148.8 (C-7), 147.4 (C-6), 137.2 (C-1′), 134.6 (C-4′), 132.9 (C-9), 127.8 (C-10), 110.2 (C-5), 109.5 (C-8), 108.0 (C-2′, C-6′), 101.7 (OCH_2_O), 68.0 (C-11), 67.5 (C-4), 62.1 (C-13^‴^), 60.7 (4′-OCH_3_), 60.3 (C-14^‴^), 56.2 (3′, 5′-OCH_3_), 55.4 (C-12^‴^), 43.8 (C-2), 41.5 (C-1), 40.4, 36.7, 34.0, 28.4, 28.1, 24.7; ESIMS: *m/z* 663 [M + Na]^+^, HRESIMS: calcd for C_32_H_36_N_2_O_10_SNa [M + Na]^+^ 663.1983, found 663.1980.

### 4β-(biotin)-4-deoxy-4′-demethylpodophyllotoxin 16

White amorphous powder, yield 59%; m.p. 153–159°C; ${\left[\alpha \right]}_{\text{D}}^{26.4}$: −69.8 (*c* 0.17, Pyridine); ^1^H-NMR (C_5_D_5_N, 500 MHz) δ 7.64 (s, 1H, C^5^-H), 7.50 (s, 1H, C^8^-H), 7.24 (s, 2H, C^2^′, C^6^′-H), 6.82 (d, 1H, *J* = 3.2 Hz, C^4^-H), 6.02–6.00 (m, 2H, OCH_2_O), 4.89 (d, 1H, *J* = 5.3 Hz, C^1^-H), 4.69 (t, 1H, *J* = 2.3 Hz), 4.54 (t, 1H, *J* = 2.1 Hz), 4.40–4.37 (m, 1H), 3.85–3.84 (m, 1H), 3.71 (s, 6H, C^3^′, C^5^′-OCH_3_), 3.22–3.19 (m, 2H), 3.92 (dd, 1H, *J* = 5.0 Hz, 10.0 Hz, C^2^-H), 2.89–2.86 (m, 2H), 2.63 (t, 2H, *J* = 7.3 Hz, C^8^^‴^-CH_2_), 1.77–1.74 (m, 2H), 1.61–1.57 (m, 2H), 1.28–1.26 (m, 2H); ^13^C-NMR (C_5_D_5_N, 125 MHz) δ 175.7 (C-12), 171.3 (C-7^‴^), 164.3 (C-16^‴^), 152.3 (C-3′, C-5′), 148.4 (C-7), 147.7 (C-6), 139.5 (C-1′), 136.1 (C-4′), 134.4 (C-9), 132.0 (C-10), 110.6 (C-5), 110.4 (C-8), 108.5 (C-2′, C-6′), 101.9 (OCH_2_O), 68.4 (C-11), 66.3 (C-4), 62.5 (C-13^‴^), 60.6 (C-14^‴^), 56.3 (3′, 5′-OCH_3_), 56.2 (C-12^‴^), 44.7 (C-2), 41.1 (C-1), 41.0, 39.7, 33.9, 29.1, 28.8, 25.4; ESIMS: *m/z* 661 [M + Cl]^−^, HRESIMS: calcd for C_31_H_34_N_2_O_10_SH [M + H]^+^ 627.2007, found 627.1978.

### 4β-(6-biotinylaminocaproic acid)-4-deoxypodophyllotoxin 17

White amorphous powder, yield 57%; m.p. 101–102°C; ${\left[\alpha \right]}_{\text{D}}^{27.0}$: −41.9 (*c* 0.13, CHCl_3_); ^1^H-NMR (CDCl_3_, 400 MHz) δ 6.84 (s, 1H, C^5^-H), 6.54 (s, 1H, C^8^-H), 6.26 (s, 2H, C^2^′, C^6^′-H), 6.14 (d, 2H, *J* = 3.2 Hz, C^4^-H), 5.99–5.98 (m, 2H, OCH_2_O), 4.66 (d, 1H, *J* = 4.8 Hz, C^1^-H), 4.52–4.50 (m, 1H), 4.36–4.32 (m, 2H), 3.87 (t, 1H, *J* = 8.8 Hz), 3.79 (s, 6H, C^3^′, C^5^′-OCH_3_), 3.73 (s, 3H, C^4^′-OCH_3_), 3.25–3.20 (m, 2H), 3.16–3.10 (m, 1H), 3.01–2.96 (m, 1H, C^3^-H), 2.89 (dd, 1H, *J* = 4.8 Hz, 12.8 Hz, C^2^-H), 2.76–2.73 (m, 2H), 2.36 (t, 2H, *J* = 8.0 Hz, C^2^^‴^-CH_2_), 2.25 (t, 2H, *J* = 6.4 Hz, C^8^^‴^-CH_2_), 1.68–1.63 (m, 6H), 1.53–1.50 (m, 2H), 1.44–1.41 (m, 2H), 1.35–1.33 (m, 2H); ^13^C-NMR (CDCl_3_, 100 MHz) δ 174.3 (C-12), 173.7 (C-1^‴^), 173.3 (C-7^‴^), 163.9 (C-16^‴^), 152.6 (C-3′, C-5′), 148.9 (C-7), 147.4 (C-6), 137.2 (C-1′), 134.6 (C-4′), 132.8 (C-9), 127.8 (C-10), 110.2 (C-5), 109.5 (C-8), 108.0 (C-2′, C-6′), 101.7 (OCH_2_O), 67.9 (C-11), 67.5 (C-4), 61.9 (C-13^‴^), 60.7 (4′-OCH_3_), 60.4 (C-14^‴^), 56.2 (3′, 5′-OCH_3_), 55.6 (C-12^‴^), 43.7 (C-2), 41.5 (C-1), 40.5, 39.3, 36.7, 35.7, 34.1, 29.1, 28.0, 27.9, 26.4, 25.7, 24.5; ESIMS: *m/z* 788 [M + Cl]^−^, HRESIMS: calcd for C_38_H_47_N_3_O_11_SCl [M + Cl]^−^ 788.2625, found 788.2627.

### 4β-(6-biotinylaminocaproic acid)-4-deoxy-4′-demethylpodophyllotoxin 18

White amorphous powder, yield 67%; m.p. 117°C; ${\left[\alpha \right]}_{\text{D}}^{25.9}$: −6.4 (*c* 0.11, CHCl_3_); ^1^H-NMR (CDCl_3_, 400 MHz) δ 6.88 (s, 1H, C^5^-H), 6.50 (s, 1H, C^8^-H), 6.29 (s, 2H, C^2^′, C^6^′-H), 5.96–5.94 (m, 2H, OCH_2_O), 4.79 (d, 1H, *J* = 3.2 Hz, C^4^-H), 4.60 (d, 1H, *J* = 5.2 Hz, C^1^-H), 4.49–4.46 (m, 1H), 4.36–4.32 (m, 2H), 4.30–4.27 (m, 1H), 3.65 (s, 6H, C^3^′, C^5^′-OCH_3_), 3.30–3.28 (m, 1H), 3.22–3.18 (m, 2H), 3.14–3.10 (m, 1H), 2.86 (dd, 1H, *J* = 5.2 Hz, 12.8 Hz, C^2^-H), 2.79–2.76 (m, 1H), 2.72–2.69 (m, 1H), 2.56 (t, 2H, *J* = 6.8 Hz, C^2^^‴^-CH_2_), 2.35–2.32 (m, 4H), 2.14 (t, 2H, *J* = 6.8 Hz, C^8^^‴^-CH_2_), 1.75–1.70 (m, 2H), 1.66–1.60 (m, 2H), 1.53–1.50 (m, 2H), 1.43–1.36 (m, 2H); ^13^C-NMR (CDCl_3_, 150 MHz) δ 175.8 (C-12), 173.8 (C-1^‴^), 171.9 (C-7^‴^), 163.9 (C-16^‴^), 151.5 (C-3′, C-5′), 148.4 (C-7), 147.5 (C-6), 138.2 (C-9), 132.3 (C-10), 131.5 (C-4′), 127.7 (C-1′), 110.4 (C-5), 109.6 (C-8), 107.7 (C-2′, C-6′), 101.7 (OCH_2_O), 68.1 (C-11), 66.3 (C-4), 62.1 (C-13^‴^), 60.5 (C-14^‴^), 56.3 (3′, 5′-OCH_3_), 55.6 (C-12^‴^), 44.1 (C-2), 40.7 (C-1), 40.5, 39.4, 38.6, 35.8, 33.7, 29.0, 28.3, 28.1, 26.2, 25.6, 24.6; ESIMS: *m/z* 738 [M – H]^−^, HRESIMS: calcd for C_37_H_45_N_3_O_11_SH [M + H]^+^ 740.2848, found 740.2809.

### 4β-amino-(biotin)-4-deoxypodophyllotoxin 19

White amorphous powder, yield 62%; m.p. 120°C; ${\left[\alpha \right]}_{\text{D}}^{26.3}$: +35.3(*c* 0.22, pyridine); ^1^H-NMR (C_5_D_5_N, 500 MHz) δ 6.90 (s, 1H, C^5^-H), 6.70 (s, 2H, C^2^′, C^6^′-H), 6.67 (s, 1H, C^8^-H), 5.96–5.93 (m, 2H, OCH_2_O), 4.84 (s, 1H, C^4^-H), 4.58 (d, 1H, *J* = 6.0 Hz, C^1^-H), 4.56–4.54 (m, 2H), 4.37–4.34 (m, 1H), 4.32–4.28 (m, 1H), 3.90 (s, 3H, C^4^′-OCH_3_), 3.69 (s, 6H, C^3^′, C^5^′-OCH_3_), 3.39 (t, 1H, *J* = 9.0 Hz), 3.17–3.12 (m, 1H, C^3^-H), 2.91 (dd, 1H, *J* = 6.0 Hz, 12.0 Hz, C^2^-H), 2.86–2.83 (m, 2H), 2.15 (t, 2H, *J* = 9.0 Hz, C^8^^‴^-CH_2_), 1.79–1.72 (m, 2H), 1.46–1.42 (m, 2H), 1.40–1.37 (m, 2H); ^13^C-NMR (C_5_D_5_N, 125 MHz) δ 178.1 (C-12), 173.2 (C-7^‴^), 164.2 (C-16^‴^), 154.2 (C-3′, C-5′), 148.0 (C-7), 146.5 (C-6), 141.3 (C-1′), 136.4 (C-4′), 129.5 (C-9), 124.1 (C-10), 112.2 (C-5), 107.2 (C-8), 107.2 (C-2′, C-6′), 101.7 (OCH_2_O), 64.2 (C-11), 62.5 (C-4), 60.7 (C-13^‴^), 60.6 (4′-OCH_3_), 56.8 (C-14^‴^), 56.4 (3′, 5′-OCH_3_), 56.3 (C-12^‴^), 51.7 (C-2), 47.2 (C-1), 42.9, 41.1, 34.1, 29.0 (2), 25.3; ESIMS: *m/z* 662 [M + Na]^+^, HRESIMS: calcd for C_32_H_37_N_3_O_9_SNa [M + Na]^+^ 662.2143, found 662.2145.

### 4β-amino-(biotin)-4-deoxy-4′-demethylpodophyllotoxin 20

White amorphous powder, yield 41%; m.p. 153°C; ${\left[\alpha \right]}_{\text{D}}^{26.0}$: −22.6 (*c* 0.10, CHCl_3_); ^1^H-NMR (CDCl_3_, 600 MHz) δ 6.82 (s, 1H, C^5^-H), 6.51 (s, 1H, C^8^-H), 6.44 (s, 2H, C^2^′, C^6^′-H), 5.98–5.94 (m, 2H, OCH_2_O), 5.62 (s, 1H, C^4^-H), 4.57 (d, 1H, *J* = 4.2 Hz, C^1^-H), 4.47–4.45 (m, 1H), 4.41 (t, 1H, *J* = 7.8 Hz), 4.28–4.26 (m, 1H), 4.16 (t, 1H, *J* = 9.6 Hz), 3.77 (s, 6H, C^3^′, C^5^′-OCH_3_), 3.14–3.10 (m, 1H), 2.90–2.86 (m, 2H), 2.75–2.70 (m, 1H, C^3^-H), 2.65–2.63 (m, 1H), 2.29–2.20 (m, 2H), 1.74–1.69 (m, 2H), 1.56–1.50 (m, 2H), 1.43–1.38 (m, 2H); ^13^C-NMR (CDCl_3_, 150 MHz) δ 175.0 (C-12), 174.2 (C-7^‴^), 164.5 (C-16^‴^), 147.7 (C-7), 147.7 (C-6), 146.6 (C-3′, C-5′), 134.7 (C-1′), 132.0 (C-4′), 131.6 (C-9), 130.7 (C-10), 110.4 (C-5), 109.2 (C-2′, C-6′), 106.6 (C-8), 101.7 (OCH_2_O), 71.8 (C-11), 69.7 (C-4), 61.9 (C-13^‴^), 60.4 (C-14^‴^), 57.1 (3′, 5′-OCH_3_), 56.1 (C-12^‴^), 46.3 (C-2), 43.9 (C-1), 40.8 39.5, 36.0, 28.5, 28.4, 25.9; ESIMS: *m/z* 660 [M + Cl]^−^, HRESIMS: calcd for C_31_H_35_N_3_O_9_SH [M + H]^+^ 626.2167, found 626.2145.

### 4β-amino-(6-biotinylaminocaproic acid)-4-deoxypodophyllotoxin 21

White amorphous powder, yield 46%; m.p. 100°C; ${\left[\alpha \right]}_{\text{D}}^{27.1}$: +58.3 (*c* 0.12, CHCl_3_); ^1^H-NMR (CDCl_3_, 400 MHz) δ 7.48 (s, 1H, NH), 6.66 (s, 1H, C^5^-H), 6.46 (s, 1H, C^8^-H), 6.29 (s, 2H, C^2^′, C^6^′-H), 5.94–5.90 (m, 2H, OCH_2_O), 4.50 (s, 1H, C^4^-H), 4.34–4.32 (m, 2H), 4.19–4.17 (m, 2H), 4.09–4.05 (m, 1H), 3.81 (s, 3H, C^4^′-OCH_3_), 3.76 (s, 6H, C^3^′, C^5^′-OCH_3_), 3.19–3.13 (m, 4H), 2.87–2.85 (m, 2H), 2.75–2.72 (m, 1H), 2.52–2.50 (m, 2H, C^2^^‴^-CH_2_), 2.24–2.23 (m, 6H), 1.69–1.64 (m, 2H), 1.51–1.48 (m, 2H), 1.45–1.42 (m, 2H), 1.29–1.24 (m, 2H); ^13^C-NMR (CDCl_3_, 100 MHz) δ 178.2 (C-12), 173.8 (C-1^‴^), 173.4 (C-7^‴^), 164.1 (C-16^‴^), 153.2 (C-3′, C-5′), 147.6 (C-7), 146.0 (C-6), 140.0 (C-1′), 136.7 (C-4′), 134.2 (C-9), 128.4 (C-10), 111.8 (C-5), 106.7 (C-8), 105.8 (C-2′, C-6′), 101.2 (OCH_2_O), 63.4 (C-11), 61.9 (C-4), 60.8 (4′-OCH_3_), 60.4 (C-13^‴^), 56.6 (C-14^‴^), 56.2 (3′, 5′-OCH_3_), 55.6 (C-12^‴^), 50.9 (C-2), 46.2 (C-1), 42.1, 40.5, 39.3, 35.6, 33.8, 28.9, 28.0, 27.9, 26.2, 25.7, 24.4; ESIMS: *m/z* 775 [M + Na]^+^, HRESIMS: calcd for C_38_H_48_N_4_O_10_SNa [M + Na]^+^ 753.3164, found 753.3154.

### 4β-amino-(6-biotinylaminocaproic acid)-4-deoxy-4′-demethylpodophyllotoxin 22

White amorphous powder, yield 64%; m.p. 100°C; ${\left[\alpha \right]}_{\text{D}}^{26.2}$: +15.1 (*c* 0.24, DMSO); ^1^H-NMR (C_2_D_6_SO, 500 MHz) δ 8.28 (s, 1H, NH), 7.78 (s, 1H, NH), 6.83 (s, 1H, C^5^-H), 6.45 (s, 1H, C^8^-H), 6.38 (s, 2H, C^2^′, C^6^′-H), 5.97–5.92 (m, 2H, OCH_2_O), 4.28 (d, 1H, *J* = 5.0 Hz, C^4^-H), 4.26 (d, 1H, *J* = 5.0 Hz, C^1^-H), 4.24 (s, 1H), 4.16 (s, 1H), 4.11–4.09 (m, 2H), 3.64 (s, 6H, C^3^′, C^5^′-OCH_3_), 3.30–3.27 (m, 2H), 3.09–3.05 (m, 1H, C^3^-H), 3.02–2.99 (m, 2H), 2.78 (dd, 1H, *J* = 5.0 Hz, 12.0 Hz, C^2^-H), 2.54–2.50 (m, 3H), 2.02 (t, 2H, *J* = 7.5 Hz, C^8^^‴^-CH_2_), 1.61–1.58 (m, 4H), 1.50–1.44 (m, 2H), 1.42–1.38 (m, 2H), 1.35–1.30 (m, 2H), 1.27–1.24 (m, 2H); ^13^C-NMR (C_2_D_6_SO, 125 MHz) δ 177.2 (C-12), 171.9 (C-1^‴^), 170.8 (C-7^‴^), 162.7 (C-16^‴^), 151.4 (C-3′, C-5′), 146.6 (C-7), 145.2 (C-6), 143.4 (C-1′), 136.1 (C-4′), 128.4 (C-9), 126.7 (C-10), 111.1 (C-5), 106.9 (C-8), 105.4 (C-2′, C-6′), 100.9 (OCH_2_O), 61.1 (C-4), 60.6 (C-11), 59.2 (C-13^‴^), 55.9 (3′, 5′-OCH_3_), 55.4 (C-14^‴^), 54.9 (C-12^‴^), 50.1 (C-2), 45.6 (C-1), 44.8, 40.0, 38.2, 35.2, 33.0, 28.8, 28.2, 28.0, 25.6, 25.3, 24.3; ESIMS: *m/z* 761 [M + Na]^+^, HRESIMS: calcd for C_37_H_46_N_4_O_10_SH [M + Na]^+^ 761.2827, found 761.2829.

### 4β-{[4-hydroxymethyl-(biotin)-1,2,3-triazol-1-yl]}-4-deoxypodophyllotoxin 23

White amorphous powder, yield 39%; m.p. 110°C; ${\left[\alpha \right]}_{\text{D}}^{27.1}$: −21.6 (*c* 0.16, CHCl_3_); ^1^H-NMR (CDCl_3_, 400 MHz) δ 7.39 (s, 1H, C^5^″-H), 6.65 (s, 1H, C^5^-H), 6.62 (s, 1H, C^8^-H), 6.32 (s, 2H, C^2'^, C^6^′-H), 6.10–6.00 (m, 3H, OCH_2_O, C^4^-H), 5.21–5.13 (m, 2H, C^6^″-CH_2_), 4.76 (d, 1H, *J* = 4.0 Hz, C^1^-H), 4.52–4.51 (m, 1H), 4.40–4.26 (m, 3H), 3.80 (s, 3H, C^4^′-OCH_3_), 3.76 (s, 6H, C^3^′, C^5^′-OCH_3_), 3.22–3.13 (m, 3H), 2.92–2.89 (m, 1H), 2.77–2.73 (m, 1H), 2.36 (t, 2H, *J* = 6.0 Hz, C^8^^‴^-CH_2_), 1.86–1.83 (m, 2H), 1.66–1.63 (m, 2H), 1.42–1.40 (m, 2H); ^13^C-NMR (CDCl_3_, 100 MHz) δ 173.6 (C-12), 173.6 (C-7^‴^), 152.7 (C-3′, C-5′), 149.4 (C-7), 148.0 (C-6), 142.9 (C-4″), 137.4 (C-1′), 134.3 (C-4′), 133.2 (C-9), 124.5 (C-10), 124.3 (C-5″), 110.5 (C-5), 108.8 (C-8), 108.1 (C-2′, C-6′), 102.0 (OCH_2_O), 67.4 (C-11), 62.0 (C-4), 60.7 (4′-OCH_3_), 60.3 (C-13^‴^), 58.7 (C-14^‴^), 57.3 (C^6^″-CH_2_), 56.3 (3′, 5′-OCH_3_), 55.4 (C-12^‴^), 43.6 (C-2), 41.5 (C-1), 40.4 37.1, 33.6, 28.1, 25.6, 24.6; ESIMS: *m/z* 744 [M + Na]^+^, HRESIMS: calcd for C_35_H_39_N_5_O_10_SNa [M + Na]^+^ 744.2310, found 744.2356.

### 4β-{[4-hydroxymethyl-(6-biotinylaminocaproic acid)-1,2,3-triazol-1-yl]}-4-deoxy-4′-demethylpodophyllotoxin 24

White amorphous powder, yield 60%; m.p. 104°C; ${\left[\alpha \right]}_{\text{D}}^{26.3}$: −60.3 (*c* 0.19, Pyridine); ^1^H-NMR (CDCl_3_, 400 MHz) δ 7.74 (s, 1H, C^5^″-H), 6.57 (s, 1H, C^5^-H), 6.45 (s, 2H, C^2^′, C^6^′-H), 6.20 (s, 1H, C^8^-H), 5.93–5.84 (m, 3H, OCH_2_O, C^4^-H), 5.23–5.15 (m, 2H, C^6^″-CH_2_), 4.65 (d, 1H, *J* = 4.0 Hz, C^1^-H), 4.46–4.44 (m, 1H), 4.22–4.16 (m, 2H), 4.11 (t, 1H, *J* = 9.2 Hz), 3.79 (s, 6H, C^3^′, C^5^′-OCH_3_), 3.30–3.25 (m, 1H), 3.03–2.99 (m, 3H), 2.79 (dd, 1H, *J* = 4.0 Hz, 10.0 Hz, C^2^-H), 2.32 (t, 2H, *J* = 6.8 Hz, C^8^^‴^-CH_2_), 1.65–1.55 (m, 4H), 1.35–1.33 (m, 2H); ^13^C-NMR (CDCl_3_, 100 MHz) δ 173.6 (C-12), 173.5 (C-7^‴^), 148.5 (C-7), 147.9 (C-6), 146.8 (C-3′, C-^5′^), 143.4 (C-4″), 134.1 (C-1′), 132.5 (C-4′), 129.9 (C-9), 126.6 (C-10), 123.1 (C-5″), 110.2 (C-5), 107.5 (C-2′, C-6′), 106.2 (C-8), 101.8 (OCH_2_O), 70.1 (C-11), 63.1 (C-4), 62.1 (C-13^‴^), 60.2 (C-14^‴^), 57.3 (C^6^″-CH_2_), 56.3 (3′, 5′-OCH_3_), 55.4 (C-12^‴^), 45.8 (C-2), 43.6 (C-1), 40.2 38.7, 33.5, 28.2, 28.1, 24.5; ESIMS: *m/z* 730 [M + Na]^+^, HRESIMS: calcd for C_34_H_37_N_5_O_10_SH [M + H]^+^ 708.2334, found 708.2302.

### 4β-{[4-hydroxymethyl-(6-biotinylaminocaproic acid)-1,2,3-triazol-1-yl]}-4-deoxypodophyllotoxin 25

White amorphous powder, yield 47%; m.p. 101°C; ${\left[\alpha \right]}_{\text{D}}^{27.2}$: −25.4 (*c* 0.24, CHCl_3_); ^1^H-NMR (CDCl_3_, 400 MHz) δ 7.41 (s, 1H, C^5^″-H), 6.62 (s, 1H, C^5^-H), 6.60 (s, 1H, C^8^-H), 6.30 (s, 2H, C^2^′, C^6^′-H), 6.11 (d, 1H, *J* = 3.2 Hz, C^4^-H), 6.00 (d, 2H, *J* = 4.8 Hz, OCH_2_O), 5.15 (s, 2H, C^6^″-CH_2_), 4.75 (d, 1H, *J* = 4.8 Hz, C^1^-H), 4.52–4.49 (m, 1H), 4.38–4.31 (m, 3H), 3.79 (s, 3H, C^4^′-OCH_3_), 3.75 (s, 6H, C^3^′, C^5^′-OCH_3_), 3.20–3.13 (m, 5H), 2.89–2.86 (m, 1H), 2.74–2.71 (m, 1H), 2.32 (t, 2H, *J* = 7.2 Hz, C^2^^‴^-CH_2_), 2.22 (t, 2H, *J* = 6.4 Hz, C^8^^‴^-CH_2_), 1.66–1.58 (m, 6H), 1.49–1.46 (m, 2H), 1.42–1.40 (m, 2H), 1.30–1.28 (m, 2H); ^13^C-NMR (CDCl_3_, 100 MHz) δ 173.7 (C-12), 173.5 (C-1^‴^), 173.3 (C-7^‴^), 164.0 (C-16^‴^), 152.7 (C-3′, C-5′), 149.4 (C-7), 148.0 (C-6), 142.9 (C-4″), 137.4 (C-1′), 134.3 (C-4′), 133.2 (C-9), 124.4 (C-10), 124.3 (C-5″), 110.5 (C-5), 108.8 (C-8), 108.1 (C-′, C-6′), 102.0 (OCH_2_O), 67.4 (C-11), 61.8 (C-4), 60.7 (4′-OCH_3_), 60.3 (C-13^‴^), 58.7 (C-14^‴^), 57.2 (C^6^″-CH_2_), 56.3 (3′, 5′-OCH_3_), 55.6 (C-12^‴^), 43.6 (C-2), 41.5 (C-1), 40.5 39.2, 37.1, 35.7, 33.8, 28.9, 28.1, 27.9, 26.1, 25.7, 24.3; ESIMS: *m/z* 857 [M + Na]^+^, HRESIMS: calcd for C_41_H_50_N_6_O_11_SH [M + H]^+^ 835.3331, found 835.3338.

### 4β-{[4-hydroxymethyl-(6-biotinylaminocaproic acid)-1,2,3-triazol-1-yl]}-4-deoxy-4′-demethylpodophyllotoxin 26

White amorphous powder, yield 58%; m.p. 119°C; ${\left[\alpha \right]}_{\text{D}}^{27.1}$: −9.6 (*c* 0.16, CHCl_3_); ^1^H-NMR (CDCl_3_, 400 MHz) δ 7.36 (s, 1H, C^5^″-H), 6.60 (s, 1H, C^5^-H), 6.59 (s, 1H, C^8^-H), 6.33 (s, 2H, C^2'^, C^6^′-H), 6.10 (d, 1H, *J* = 4.0 Hz, C^4^-H), 5.99–5.96 (m, 2H, OCH_2_O), 4.75 (s, 1H, C^1^-H), 4.68 (s, 2H, C^6^″-CH_2_), 4.49–4.46 (m, 1H), 4.37–4.36 (m, 1H), 4.30–4.27 (m, 2H), 3.67 (s, 6H, C^3^′, C^5^′-OCH_3_), 3.20–3.10 (m, 6H), 2.85 (dd,1H, *J* = 4.8 Hz, 12.2 Hz, C^2^-H), 2.64 (m, 2H, *J* = 7.2 Hz, C^2^^‴^-CH_2_), 2.56 (t, 2H, *J* = 6.8 Hz, C^8^^‴^-CH_2_), 1.73–1.68 (m, 2H), 1.66–1.58 (m, 4H), 1.53–1.50 (m, 2H), 1.45–1.35 (m, 4H); ^13^C-NMR (CDCl_3_, 125 MHz) δ 174.0 (C-12), 173.6 (C-1^‴^), 171.7 (C-7^‴^), 164.0 (C-16^‴^), 151.6 (C-3′, C-5′), 149.4 (C-7), 148.1 (C-6), 148.0 (C-4″), 137.2 (C-1′), 132.8 (C-4'), 128.0 (C-9), 124.7 (C-10), 122.9 (C-5″), 110.5 (C-5), 108.9 (C-8), 107.6 (C-2′, C-6′), 102.0 (OCH_2_O), 67.5 (C-11), 62.1 (C-4), 60.4 (C-13^‴^), 58.7 (C-14^‴^), 56.1 (3′, 5′-OCH_3_), 55.6 (C^6^″-CH_2_), 55.4 (C-12^‴^), 43.6 (C-2), 41.4 (C-1), 40.1, 39.3, 37.0, 35.4, 33.5, 28.7, 28.1, 27.8, 26.0, 25.5, 24.4; ESIMS: *m/z* 843 [M + Na]^+^, HRESIMS: calcd for C_40_H_48_N_6_O_11_SH [M + H]^+^ 821.3175, found 821.3195.

### Biology Assay

#### Cell Culture

All cell lines used in this study were cultured in DMEM or RMPI-1640 medium (Hyclone, Logan, UT, USA) which is supplemented with 50 mg/L of streptomycin, 50 IU/ml of penicillin (Solarbio, Beijing, China) and 10% fetal bovine serum (HyClone, CA, USA) in a humidified 5% CO_2_ incubator at 37°C. All cells were sub-cultured 3 times/week by trypsinisation.

### Cell Viability Assay

Cell viability was evaluated by 3-(4,5-dimethyl-thiazol-2-yl)-2,5-diphenyltetrazolium bromide (MTT) assay. Briefly, in each well of a 96-well cell culture plate adherent cells (100 μL) with an initial density of 1 × 10^5^ cells/mL were seeded and allowed to adhere for 12 h before a test drug was added. In contrast, suspended cells with the same initial density were seeded just before drug addition. Each tumor cell line was exposed to the test compound at various concentrations in triplicate for 48 h. After the incubation, MTT (100 μg) was added to each well, and the incubation continued at 37°C for 4 h. The cells were lysed with SDS (200 μL) after the removal of the medium. The absorbance of the lysate was measured at 595 nm by spectrophotometry (microtiter plate reader, Bio-Rad 680). Dose response curves of cell viability were plotted and the IC_50_ values of test compounds at which 50% reduction in cell growth were determined.

### Cell Apoptosis Assay

The Annexin V/propidium iodide (PI) detection kit (BD Biosciences, PA, USA) was employed to quantify apoptosis using flow cytometry. H1299 and H1975 cells were seeded in each well of a 12-well plate at 2.5 × 10^5^ cells/well. After incubation for 24 h, the cells were treated with compound **15** at 0.5, 1 and 2 μM or **PPT** (1 μM) for 24 h. Then, the cells were collected and binding buffer (100 μL), FITC annexinV (5 μL), and propidium iodide (PI, 10 μL) (eBioscience, San Diego, CA, USA) were added to the cell suspension. The cells were gently vortexed and incubated at room temperature in the dark for 15 min before measurement by flow cytometry (BD FACSCalibur^TM^) within 1 h.

### Western Blotting Analysis

H1299 and H1975 cell lines were treated with compound **15** at different concentrations in 6-well plates, and then the cells were collected and lysed with lysis buffer. After sonication cells were centrifuged at 14,000 rpm at 4°C for 10 min, and total protein was extracted and detected using a bicinchoninc acid (BCA) assay kit. The samples were then separated by 10% SDS-polyacrylamide gel electrophoresis (PAGE) and then the protein was transferred to nitrocellulose (NC) membranes. The membranes were probed for the following proteins with primary antibodies at 4°C overnight: caspase-3, cleaved caspase-3, PARP, cleaved PARP, GRP78, CHOP, XBP-1, XBP-1s, and Actin. After washing the membranes with PBST (× 1), the HRP-conjugated secondary antibodies were added and incubated for 1 h at room temperature. The membranes were then washed and the HRP was detected using Luminata™ Forte Western HRP Substrate reagent. The bands of interest were visualized and imaged under chemiluminescent detection using a FluorChem E System (ProteinSimple, San Jose, CA, USA).

### Gene Expression Assay

H1299 cells were cultured in 12-well plated at 2.5 × 10^5^ cell/well in the presence of compound **15** (0.5, 1, and 2 μM) for 24 h. Total RNAs present in the cultured cells were extracted using the TransZol™ Up Reagent (TransGen Biotech, Beijing, China). Gene expression was detected via quantitative real-time PCR (qRT-PCR) and SYBR® Premix EX Taq™ II (TaKaRa Bio, Otsu, Japan) was used to perform the analysis.

### Animal Studies

All animal studies were conducted in accordance to procedures approved by the Animal Care and Use Committee at China Pharmaceutical University (Jiangsu, China). Forty icr male mice (10–20 g) were provided from the Comparative Medicine Centre of Yangzhou University (Jiangsu, China) and were housed in an SPE animal facility. S180 cancer cells were injected subcutaneously into the right axillaries of icr male mice (4.5–5.0 × 10^6^ cells/spot). The mice were divided randomly into five groups: model; positive control; low-dose; medium-dose; high-dose. All mice of the therapeutic groups were injected intraperitoneally (i.p.) every day, and all mice in the positive control group were injected intravenously (i.v.) on the first day and the fourth day after inoculation. Tumor size was measured with caliper and the volume calculated using the previously reported method (Qin et al., 2015). The weight of the mice and the volume of the tumors were measured every day. At the end of the experiment, the mice were killed and the tumors were isolated and weighed.

### Molecular Docking Studies

The crystal structure of Top-II (code ID: 3QX3) (Wu et al., 2011) was obtained in Protein Data Bank after eliminating the inhibitor and water molecules. The missing atoms were added by Sybyl-X 2.0 molecular modeling. The kinds of atomic charges were taken as Kollman-united-atom (Weiner et al., 1984) for the macromolecule and Gasteiger–Marsili (Gasteiger and Marsili, 1980) for the inhibitor. To find the binding mode of compound **15** to the active site of Top-II, the advanced docking program Autodock Tools v1.56 (Morris et al., 1998) was used for grid and docking. The enzyme structure was used as an input for the AutoGrid program. AutoGrid performed pre-calculated atomic affinity grid maps for each atom type in the ligand plus a separate desolvation map, and a separate desolvation map present in the substrate molecule. Docking parameters were set as the default values except docking runs was set to 100 on AutoGrid v4.01 and AutoDock v4.01.

### Statistical Analysis

All data are presented as the means ± SD (*n* = 3). Significance was calculated using Student's *t*-test or one-way ANOVA. *P* < 0.05 was considered statistically significant. All statistical analyses were performed with the GraphPad Prism 5.0 (San Diego, CA, USA).

## Results and Discussion

### Chemical synthesis

**Scheme 1:**
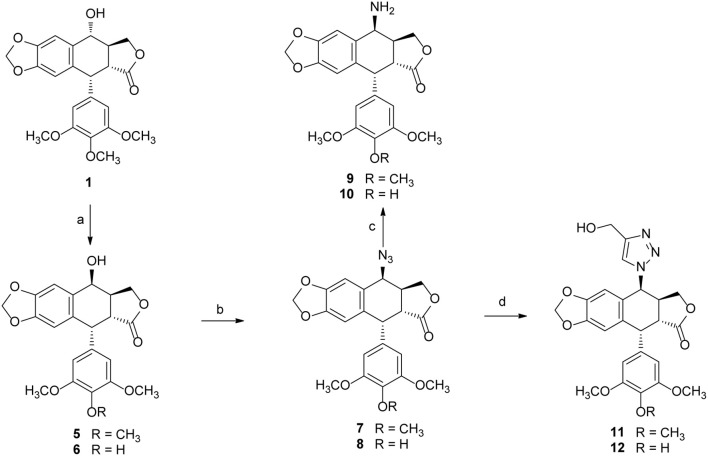
The synthesis of podophyllotoxin derivatives (**5**–**12**). Reagents and reaction condition: (a) MeSO_3_H, NaI, CH_3_CN/CH_2_Cl_2_; then, H_2_O-Acetone, BaCO_3_, rt. 90–92%; (b) NaN_3_-TFA, CHCl_3_, 67–70%; (c) PPh_3_, THF, then H_2_O, 72–75%; (d) copper (II) acetate, propargyl alcohol, sodium ascorbate, *t*-BuOH-H_2_O, THF, rt. 85–89%.

**Scheme 2:**
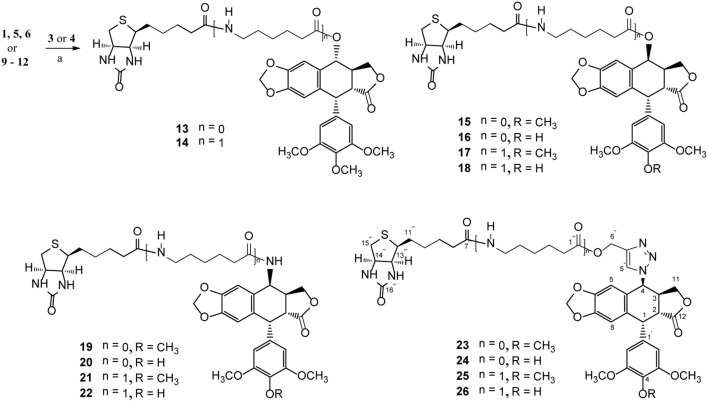
The synthesis of biotinylated podophyllotoxin derivatives (**13**–**26**). Reagents and reaction condition: (a) DIC, DMAP, DMF, N_2_, rt. 39–65%.

Podophyllotoxin (**PPT**) served as the starting material for the preparation of all the derivatives. The incorporation of the azido, amino, and triazolyl groups at the 4-position of **PPT** followed standard procedures (Scheme 1). **PPT** was regioselectively demethylated with methanesulfonic acid and sodium iodide in dichloromethane (CH_2_Cl_2_) followed by weak basic hydrolysis (water-acetone, barium carbonate) to give 4′-*O*-demethylepipodophyllotoxin **6** by means of a previously described procedure (Kamal et al., 2000). When the reaction was carried out in acetonitrile (CH_3_CN) as a solvent, 4*β*-epipodophyllotoxin **5** was synthesized as product. Compound **5** and **6** were converted into the corresponding 4*β*-azides **7** and **8** by reaction with sodium azide and trifluoroacetic acid (NaN_3_-TFA) in chloroform (CHCl_3_) according to the known procedure (Hansen et al., 1993). The 4*β*-azides **7** and **8** were converted to 4*β*-amino substituted **9** and **10** by treatment with triphenylphosphine (Ph_3_P) and water overnight at 25°C as previously reported (Coleman and Kong, 1998). In addition, the 4*β*-triazole compounds of **11** and **12** were prepared in 85–89% yield by the reaction of **7** and **8**, respectively, with 2-propyn-1-ol using copper (II) acetate and sodium ascorbate as promoters in *tert*-butanol and water (*t*-BuOH-H_2_O, 1:1) at room temperature (Tae et al., 2010). Finally, biotin (**3**)/6-biotinylaminocaproic acid (**4**) and those podophyllotoxin derivatives (**1**, **5**, **6**, and **9**–**12**) were coupled via an ester or amide bond. As shown in Scheme 2, biotin (**3**)/6-biotinylaminocaproic acid (**4**) reacted with compounds **1**, **5**, **6**, and **9**–**12** in the presence of diisopropylcarbodiimide (DIC) and 4-*N*,*N*-dimethylaminopyridine (DMAP) at room temperature to afford the target compounds **13**–**26** in 39–65% yields.

All the products were structurally confirmed by ^1^H and ^13^C-NMR spectroscopies, as well as low resolution and high resolution mass spectrometry in electrospray ionization mode (ESI-MS and HRESI-MS). The proton and carbon-13 NMR data of these compounds were compared with those of podophyllotoxin. The configuration of C-4 in compounds **13**–**26** was assigned based on the coupling constant between H-3 and H-4 (*J*_3, 4_). Typically, compounds with C-4*β*-substitution have *J*_3, 4_ <5.0 Hz as a result of H-3 and H-4 in *cis* relationship. The protons at C-4 of compounds **19**–**21** appear as a singlet. On the other hand, compounds with C-4α-substitution have *J*_3, 4_ > 6.0 Hz because H-4 is *trans* to H-3 (Fred Brewer et al., 1979; Belen'kiib and Schinazi, 1994). In the ^13^C-NMR spectra, the C-4 of these derivatives produces a characteristic signal between 61.1 and 71.3 ppm. The triazole ting in **23**–**26** was readily confirmed by its C^5“^-H signal (δ 7.36–7.74 ppm) in the aromatic region in the ^1^H-NMR spectra, which was further supported by the characteristic carbon signals at around 123 ppm in the ^13^C-NMR spectra.

### Biology

#### Cytotoxicity and Structure-Activity Relationship

The cytotoxicity of all biotinylated podophyllotoxin derivatives **13**–**26** was tested with the following cancer cell lines: SW480 (colon cancer), MCF-7 (breast cancer), A-549 (lung cancer), SMMC-7721 (hepatoma), and HL-60 (leukemia), Podophyllotoxin (**PPT**), etoposide, and cisplatin were included for study as control drugs. The IC_50_ values obtained from MTT assay are presented in Table 1. Most compounds possessed high level of cytotoxicity against all five cancer cell lines (Table 1) and were more active than etoposide which is an antitumor agent currently in clinical use.

**Table 1:** The *in vitro* cytotoxic activity (IC_50_, μM) of biotinylated podophyllotoxin derivatives **13** – **26** and **PPT**.

**Compds**.	**IC**_****50****_ **(μM)**				
	**HL-60**	**SMMC-7721**	**A-549**	**MCF-7**	**SW480**
**13**	0.22	0.75	0.73	1.48	1.46
**14**	0.18	0.65	0.58	1.26	0.96
**15**	0.13	0.23	0.51	0.84	0.56
**16**	0.18	0.72	1.19	8.00	0.79
**17**	0.19	0.71	0.80	2.97	>40
**18**	0.21	0.70	0.67	0.85	3.09
**19**	13.94	19.28	28.99	17.57	27.13
**20**	0.46	1.40	1.34	1.60	1.25
**21**	13.83	31.23	24.37	16.94	>40
**22**	>40	>40	>40	>40	>40
**23**	10.38	19.21	17.33	23.46	39.9
**24**	3.64	>40	30.67	>40	>40
**25**	>40	>40	>40	>40	>40
**26**	>40	>40	>40	>40	>40
**PPT**	<0.064	4.13	<0.064	<0.064	9.42
Etoposide	0.31	8.12	11.92	32.82	17.11
Cisplatin	1.17	6.43	9.24	15.86	13.42

Biotinylated podophyllotoxin derivatives are prepared by linking a biotinylating agent, biotin (**3**) or 6-biotinylaminocaproic acid (**4**), via an ester bond, an amide bond, or a trizolyl moiety. Those compounds with an ester linkage (**13**–**18**) display potent cytotoxicity with IC_50_ values in sub-μM to low μM (except compound **17** against SW480 cell line). Compounds **13** and **14** are esters of podophyllotoxin while **15**–**18** are esters of 4-epipodophyllotoxin, and their similar potency of activity indicates that the cytotoxic activity of these compounds is not much affected by the configuration of C-4. Among the synthesized compounds, compound **15** is the most active one with IC_50_ ranged from 0.13 to 0.84 μM. Compound **15** also exhibits higher activity than **PPT** in both SMMC-7721 and SW480 cell lines, with **PPT** having IC_50_ of 4.13 and 9.42 μM, respectively.

Compounds with an amide linkage (**19**–**22**) or a triazolyl moiety (**23**–**26**) show weaker cytotoxicity to all tested cell lines. Most of these compounds display moderate (IC_50_ > 10.36 μM) to very weak activity (IC_50_ > 40 μM; except compound **20**, as well as compound **24** against HL-60 cell line). The 6-aminocaproic acid linking spacer present in C-4-substituent can affect the cytotoxic potency of these compounds but not in a uniform way. For example, compounds lacking the linking spacer (**15**, **20**, and **23**) show higher activity than their counterparts bearing the linking spacer (**17**, **22**, and **25**) in all cell lines tested. In contrast, compound **13** (lacking the linking spacer) is less active than **14** (bearing the linking spacer). In most cases, the effect of 6-aminocaproic acid linking spacer on the cytotoxic potency of these compounds are relatively small except for the pair of compounds **15** and **17** in SW480 cell line (IC_50_ 0.56 and > 40 μM, respectively). However, it is very interesting to note that compound **20** (lacking the linking spacer, IC_50_ 0.46–1.60 μM) is significantly more potent than **22** (bearing the linking spacer, IC_50_ > 40 μM) in all cell lines tested. Furthermore, compound **19**, the 4'-*O*-methylated form of **20**, shows much lower activity (IC_50_ 13.94–28.99 μM) than **20**, which is in good agreement with our earlier observation that the 4'-*O*-methylation can significantly affect the anticancer activity of podophyllotoxin derivatives (Zi et al., 2015, 2017).

### Compound 15 Inhibits the Growth of Cancer Cell Lines

To further identify the anticancer effect and tumor selectivity of compound **15**, we treated 12 more human cancer cell lines with compound **15**, which included lung cancer (H460, H1975, H1299), colon cancer (LS174T, HCT-116, HT-29), stomach cancer (BGC-823, MGC-803), breast cancer (SKBR3, T47D), hepatoma (Bel-7402), and cervical cancer (Hela). MTT assay was employed to provide the IC_50_ values of compound **15** against all these tumor cell lines as shown in Table 2. H1299 cell line was most sensitive toward compound **15** (IC_50_ = 0.86 μM). For most other cancer cell lines, compound **15** showed potent anticancer activity with IC_50_ values in μM range. In order to test whether compound **15** can favorably target cancer cells over normal cells, the growth inhibitory effect of **15**, in comparison with **PPT**, on a normal human bronchial epithelial cell line (BEAS-2B) was evaluated. The IC_50_ value was found to be 3.75 μM for **15** and 0.85 μM for **PPT** against BEAS-2B cells (see Table S1). Comparing with its IC_50_ values in Table 1 (0.13–0.84 μM) and Table 2 against various cancer cell lines, compound **15** does show some selectivity against certain tested cancer cell lines over the normal cells (BEAS-2B).

**Table 2:** IC_50_ values of compound **15** in twelve cancer cell lines.

**Cell line**	**Type**	**IC_**50**_ (μM)**
H460	Lung cancer	6.53
H1975	Lung cancer	3.31
H1299	Lung cancer	0.86
LS174T	Colon cancer	3.30
HCT-116	Colon cancer	1.08
HT-29	Colon cancer	1.39
BGC-823	Stomach cancer	4.41
MGC-803	Stomach cancer	4.45
SKBR3	Breast cancer	5.93
T47D	Breast cancer	>10
Bel-7402	Hepatoma	10.0
Hela	Cervical cancer	1.04

### Compound 15 Induces Apoptosis in the H1299 and H1975 Cell Lines

**Figure 2:**
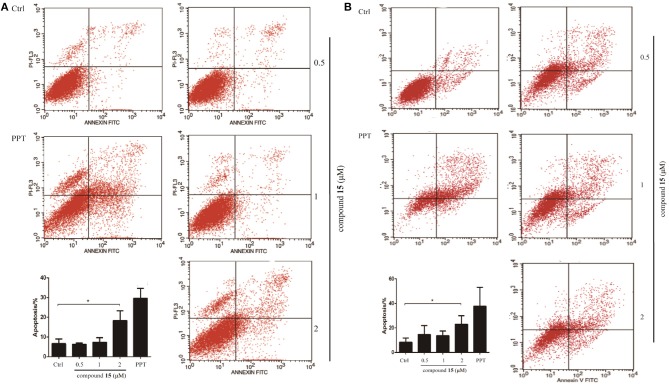
Flow cytometry analysis of lung cancer cell lines H1299 and H1975 after treatment with compound **15**. Cells were treated with compound **15** (0.5, 1, and 2 μM) and **PPT** (1 μM) for 24 h, then stained with Annexin V/7AAD and analyzed by flow cytometry. The ratio of apoptotic cells in each group was expressed as percentage. **(A)** H1299 cell line; **(B)** H1975 cell line. The data are presented as the mean ± SD (*n* = 3). *p < 0.01.

Given that compound **15** exhibits broad spectrum inhibitory activity of cancer cell growth, we studied further the capacity of compound **15** in the induction of cell death through apoptosis. Lung cancer cells (H1299 and H1975) were treated with compound **15** and analyzed by flow cytometry after being stained with Annexin V/7AAD. Compound **15** at concentration of 2 μM increased significantly both H1299 and H1975 cells undergoing apoptosis when compared with the untreated control (Figure 2).

### Compound 15 Regulates the Expression Levels of Apoptosis-Related Protein

**Figure 3:**
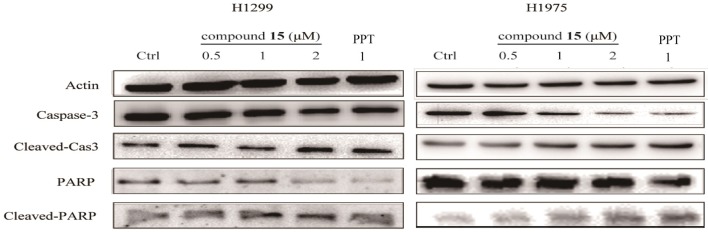
Compound **15** regulates the expression levels of apoptosis-related proteins: H1299 and H1975 cell lines were treated with compound **15** (0.5, 1, and 2 μM) and **PPT** (1 μM) for 24 h and the expression level of caspase-3, PARP, cleaved-caspase-3, and cleaved-PAPR was detected by western blot (WB). Actin was tested as a loading control.

It has been recognized that caspase-3 and PARP (poy ADP ribose polymerase) is a critical initiator and executioner of apoptosis (Hensley et al., 2013). H1299 and H1975 cells were treated with compound **15** at the concentration of 0.5, 1, 2 or μM for 24 h and the expression level of caspase-3, PARP, cleaved-caspase-3, and cleaved-PARP was monitored using western blot. The treatment of both H1299 and H1975 cell lines with compound **15** resulted in an increased expression level of cleaved-caspase-3 and cleaved-PARP in a dose-dependent manner (Figure 3). At the same time, the expression level of caspase-3 and PARP decreased, indicating that the treatment led to the activation of caspase-3 and the deactivation of PARP and ultimately apoptosis. These data confirmed that the compound **15** exhibits its anticancer activity through induction of apoptosis in both H1299 and H1975 cell lines.

### Compound 15 Induces Apoptosis Through Activating IRE1α, a Key Mediator in the Endoplasmic Reticulum (ER) Stress Pathway

**Figure 4:**
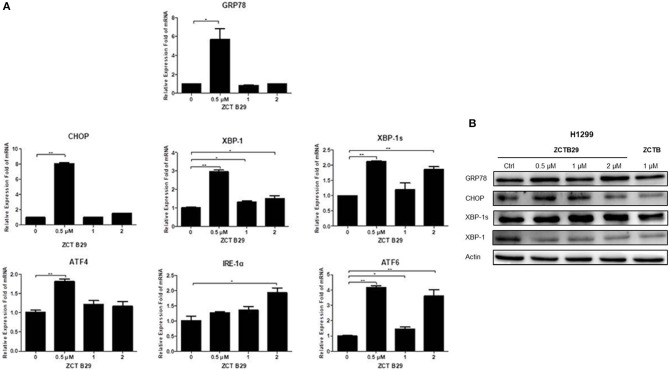
Compound **15** induces apoptosis through activating the ER stress pathway: **(A)** H1299 cell line was treated with compound **15** (0.5, 1, and 2 μM) for 24 h, GRP78, CHOP, XBP-1, XBP-1s, ATF4, IRE-1α, and ATF6 were measured by real-time RT-PCR. The data are presented as the mean ± SD (*n* = 3). *p < 0.01, **p < 0.001, ***p < 0.0001 and ****p < 0.00001. **(B)** H1299 cell line was treated with compound **15** (0.5, 1, and 2 μM) and **PPT** (1 μM) for 24 h, and WB was performed to detect the expression levels of protein in the ER stress pathway. Actin was tested as a loading control.

Many studies have indicated that endoplasmic reticulum (ER) stress activates the unfolded protein response (UPR), through which tumor cells can become resistant to chemotherapeutic agents (Cheng et al., 2014). PKR-like ER kinase (PERK), inostitol-requiring transmembrane kinase and endonuclease 1α (IRE-1α), and activating transcription factor 6 (ATF6) are three primary UPR sensors that lead to distinct downstream signaling pathways (Ron and Walter, 2007). Therefore, we next studied the possible involvement of compound **15** in the activation of the ER stress pathway. H1299 cell line was treated with compound **15** at the concentration of 0.5, 1, or 2 μM for 24 h and the mRNA expression level of stress related proteins (GRP78, CHOP, XBP-1, XBP-1s, ATF4, IRE-1α, and ATF6) in ER was analyzed (Figure 4A). Interestingly, the mRNA level of all these proteins except IRE-1α was dramatically increased upon the treatment of compound **15** at 0.5 μM. The effect of **15** at other concentrations (1 or 2 μM) on the mRNA expression level of these proteins was less significant or negligible. In the case of IRE-1α, the expression level of mRNA increased by the treatment of **15** in a dose-dependent manner, suggesting that IRE-1α might play a crucial role in compound **15**-induced apoptosis. We further examined the expression level of a number of these proteins (GRP78, CHOP, XBP-1, and XBP-1s) related to ER stress (Figure 4B). Compound **15** significantly up-regulated the expression of GRP78 and XBP-1s, and down-regulated the expression of CHOP and XBP-1.

### Compound 15 Significantly Inhibits the Growth of S180 Tumor Xenografts in Icr Mice

**Figure 5:**
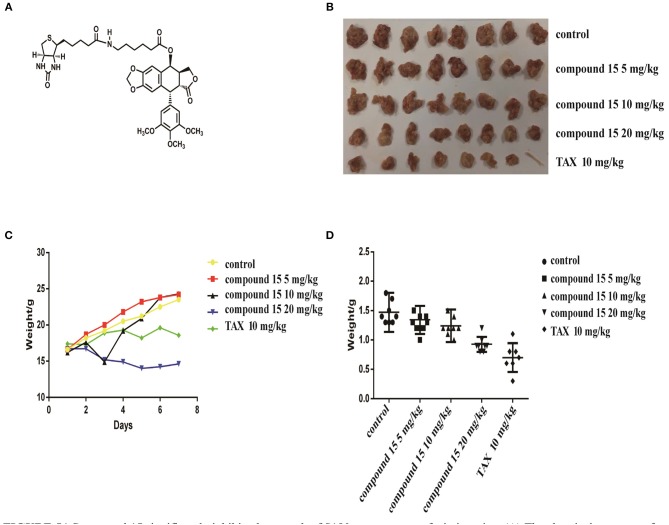
Compound **15** significantly inhibits the growth of S180 tumor xenografts in icr mice: **(A)** The chemical structure of compound **15**. **(B)** Tumors collected at the end of the treatment (day 7). **(C)** Mouse's body weights were weighed for those treated with compound 15 (5, 15, 20 mg/kg) compared to TAX control (10 mg/kg) TAX: Taxol. **(D)** Tumor weights were presented for the groups treated with compound 15 (5, 15, 20 mg/kg).

Since compound **15** suppressed lung cancer cell proliferation *in vitro*, we further investigated its ability to suppress the growth of S180 tumor xenografts in icr mice (Figure 5). As shown in Figure 5, compound **15** (5, 15, or 20 mg/kg) suppressed the growth of S180 xenografts over the course of 7 days (Figure 5A) comparing to the Taxol® control (10 mg/kg) and the inhibition rates of compound **15** were 8.8, 15.7, and 37.7% (Figure 5D), respectively. Tumors were collected at the end of the experiment (Figure 5B) and the tumor weights were measured. The data showed that compound **15** significantly decreased tumor weight when compared to the untreated control, indicating that **15** effectively inhibited the growth S180 tumor xenograft. In addition, compound **15** did not cause mice to die and did not affect mouse body weight significantly at a dose of up to 20 mg/kg (Figure 5C).

### Docking Studies

**Figure 6:**
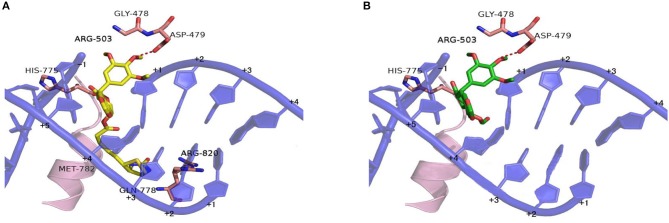
Proposed binding models of compounds **15** and **PPT** to the ATPase domain of Topoisomerase-II. Based on the X-ray co-crystal structure of Top-II in complex with Etoposide. **(A)** Binding mode of compound **15** with Topoisomerase-II. **(B)** Binding mode of **PPT** with Topoisomerase-II.

Based on the X-ray crystal structure of Topoisomerase-II inhibitors bound to the ATPase domain of Topo-II (PDB: 3QX3) (Wu et al., 2011), the binding mode between Topoisomerase-II and **15** or **PPT** was established by autodocking (Figure 6). Compound **15** binds Topo-II between the base pairs immediately flanking the two cleaved scissile phosphates (Figure 6A). Its polycyclic podophyllotoxin core (rings A to D) sits between base pairs, while the biotin side chain and the E ring protrude toward the DNA major and minor grooves, respectively. All parts of the podophyllotoxin core contribute to drug-DNA interaction by being located between base pairs. The E ring is anchored by both interacting with GLY-478, ASP-479, and ARG-503 residues of the enzyme and being sandwiched between R503 and the deoxyribose ring of the +1 nucleotide. Compared to **PPT** (Figure 6B), compound **15** shows additional hydrophobic interaction with GLN-778 and ARG-820 residues through the biotin moiety. The biotin moiety in **15** provides an additional hydrophobic moiety and multiple H-bond donors/acceptor, which allows the molecule to interact more favorably with Topo-II and might lead to improved selectivity.

### Chemical Stability Investigation

The chemical stability of compound **15** in aqueous phase was investigated together with podophyllotoxin (**PPT**, **1**) for comparison. The results indicate that compound **15** degrades slowly under the physiological condition (37 ± 1°C, pH 7.0) with 70% material remaining after 12 h (see Figures S1–S3). A similar stability profile was observed for **PPT** with 75% material remaining after 12 h.

## Conclusion

In summary, a series of biotinylated podophyllotoxin derivatives (**13**–**26**) were designed, synthesized, and evaluated for cytotoxicity against five tumor cell lines (HL-60, SMMC-7721, A-549, MCF-7, and SW480) by using MTT assay. Among them, compound **15** showed the highest anticancer activity with its IC_50_ values at 0.13–0.84 μM. Preliminary structure-activity relationship (SAR) analysis indicated that derivatives bearing an amide or triazolyl linking moiety showed weaker activity than those with an ester linkage. The 6-aminocaproic acid linking spacer affected the cytotoxic potency of these compounds in an ununiform manner. Compound **15** also reduced the expression levels of caspase-3 and PARP. Importantly, the pro-apoptotic activity of compound **15** in H1299 cell line was mediated by the transcription of IRE-1α, which plays an important role in the endoplasmic reticulum stress pathway. Finally, compound **15** at a dose of 20 mg/kg suppressed the growth of S180 tumor xenografts in icr mice significantly. Molecular docking studies suggested that compound **15** could bind well with the ATPase domain of Topoisomerase-II. Continuing studies to substantiate the further development of compound **15** as an anticancer agent are underway in our laboratory and will be reported in due course.

## Data Availability

This manuscript contains previously unpublished data. The name of the repository and accession number are not available.

## Author Contributions

JZ, J-MH, and Z-HJ designed and guided this study. C-TZ and F-WD conducted the chemical synthesis. LY and YL performed the cell assay. YH and F-QX participated in the cell assay. S-TY, Y-SG, and S-YF performed animal experiments. YJ performed molecular docking. LS and Z-TD performed the SPR binding assay. LS, Z-TD, and J-MH contributed reagents, materials, and analysis tools. C-TZ and Y-SG analyzed the data. C-TZ, LY, Y-SG, Z-HJ, and J-MH wrote the manuscript. All authors read and approved the final manuscript.

### Conflict of Interest Statement

The authors declare that the research was conducted in the absence of any commercial or financial relationships that could be construed as a potential conflict of interest.
