# Supplementary material for: Design, Synthesis, and Biological Evaluation of Novel Biotinylated Podophyllotoxin Derivatives as Potential Antitumor Agents
Source: Front Chem. 2019 Jun 18;7:434. doi: 10.3389/fchem.2019.00434 (PMC6596340; doi:10.3389/fchem.2019.00434)
Supplement: Supplementary file 1 [file Data_Sheet_1.doc]

**Supporting Information**

- **The cytotoxicity of compound 15 on normal cells**

**TABLE S1 |** IC_50_ values of compound **15** on normal cells (BEAS-2B).

| **Compounds** | IC**_50_ (**μM**)** |
| --- | --- |
|  | BEAS-2B |
| **15** | 3.75 |
| **PPT** | 0.85 |
| **Cisplatin** | 9.07 |

- **Investigation of chemical stability**

*1. Materials*

Methanol was used as chromatography grade. Phosphate buffer (0.1 M, *p*H 7.0) was prepared with Na_2_HPO_4_·12H_2_O (1.110 g) and NaH_2_PO_4_·2H_2_O (0.296 g) in deionized water (50 mL). Compounds **1** and **15** were prepared according to the procedures described in the experimental section, and purified prior to use.

*2. HPLC conditions*

Analytic HPLC was performed on an Agilent 1260 liquid chromatography with an XTerra RP_18_ (4.6 × 250 mm) column. The detecting wavelength was 254 nm. The injecting volume was 10 *µ*L. The flow rate was 1mL/min. Mixed solvents of methanol and water (55% for compound **1**, 80% for compound **15**, respectively) were used as the mobile phase.

*3. Methods*

Compounds **1** and 1**5** (2 mg each) were dissolved in acetonitrile (2 mL) for reserve. Transfer the solution (0.1 mL) to a flask, and phosphate buffer (0.1 M, 0.9 mL) was added to obtain the testing solution (0.1 mg/mL). The resulting solution was immersed in water bath at 37 ± 1℃, and analyzed by HPLC at the time point of 0 h, 1 h, 2 h, 4 h, 8 h and 12 h. Assuming the peak area of the compound at 0 h were 100%, calculate the percentage of the major component remain in the solution at each time point. Then a curve was drawn to illustrate the chemical stability of the tested compound. The chromatograms of compounds **1** and **15** are demonstrated in Figures S2 – S3. And a diagram of a comparison of chemical stability of compounds **1** and **15** was shown in Figure S4.

0h

1h

2h

4h

8h

12h

**FIGURE** **S1 |** RP-HPLC chromatograms of compound **1** kept in phosphate buffer (0.1M, *p*H 7.0) at 37 ℃ for a specific time (0h, 1h, 2h, 4h, 8h, 12h).

0h

1h

2h

4h

12h

8h

**FIGURE** **S2 |** RP-HPLC chromatograms of compound **15** kept in phosphate buffer (0.1 M, *p*H 7.0) at 37 ℃ for a specific time (0h, 1h, 2h, 4h, 8h, 12h).


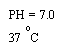

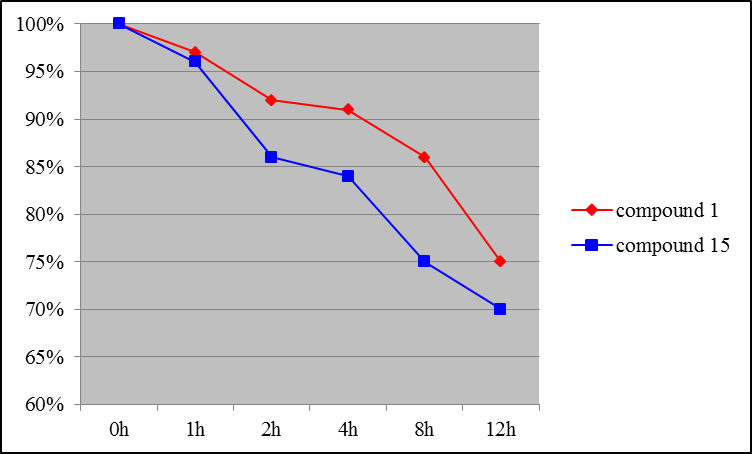


**FIGURE** **S3 |** Summary of chemical stability investigation of compounds **1** and **15.**
